# Green synthesis of NiO and NiO@graphene oxide nanomaterials using *Elettaria cardamomum* leaves: Structural and electrochemical studies

**DOI:** 10.1016/j.heliyon.2024.e38613

**Published:** 2024-09-30

**Authors:** Ayesha Kiran, Shabbir Hussain, Israr Ahmad, Muhammad Imran, Muhammad Saqib, Bushra Parveen, Khurram Shahzad Munawar, Wissem Mnif, Maryam Al Huwayz, Norah Alwadai, Munawar Iqbal

**Affiliations:** a Department of Chemistry, Khwaja Fareed University of Engineering and Information Technology, Rahim Yar Khan, 64200, Pakistan; b Division of Inorganic Chemistry, Institute of Chemistry, The Islamia University of Bahawalpur, Bahawalpur, 63100, Pakistan; c Department of Chemistry, Government College University Faisalabad, Pakistan; d Institute of Chemistry, University of Sargodha, 40100, Pakistan; e Department of Chemistry, University of Mianwali, 42200, Pakistan; f Department of Chemistry, Faculty of Sciences at Bisha, University of Bisha, P.O. BOX 199, Bisha, 61922, Saudi Arabia; g Department of Physics, College of Sciences, Princess Nourah bint Abdulrahman University, P.O. Box 84428, Riyadh, 11671, Saudi Arabia; h School of Chemistry, University of the Punjab, Lahore, 54590, Pakistan

**Keywords:** Green synthesis, NiO, Graphene oxide, Nanomaterials, Electrochemical

## Abstract

An eco-friendly synthetic route was developed for the formation of nickel oxide (NiO_aq_ and NiO_et_) nanoparticles (NPs) by treating Ni(NO_3_)_2_.6H_2_O with aqueous/ethanolic extracts of *Elettaria cardamomum* leaves; the same reaction was performed in the presence of graphene oxide (GO) to produce NiO_aq_@GO and NiO_et_@GO nanocomposites (NCs), respectively. The NMs were characterized by XRD, FT-IR, SEM, EDX, UV–visible spectroscopy, and TGA-DSC analysis. They were also subjected to electrochemical investigations and photocatalytic degradation of crystal violet (CV) dye. XRD analysis revealed the average crystallite sizes of 8.84–14.07 nm with a face-centered cubic form of NiO NPs and a hexagonal structure of their nanocomposites with GO. FT-IR spectroscopy confirmed the presence of Ni-O vibrations at 443-436 cm^−1^. SEM images confirmed the spherical morphology of NiO NPs while NiO_aq_@GO NCs contained randomly aggregated, thin, and wrinkled graphene sheets. NiO_aq_ and NiO_et_ have shown particle sizes of 27.7–30.63 nm which were decreased to 19.33–26.39 nm in their respective NiO_aq_@GO and NiO_et_@GO NCs. EDX spectra verified the homogeneous distribution of elements (Ni, O, C) on the surface of the particles. The synthesized NCs have shown smaller band gaps (NiO_aq_@GO = 3.74 eV; NiO_et_@GO = 3.34 eV) as compared to their respective NPs (NiO_aq_ = 5.0 eV; NiO_et_ = 3.89 eV). TGA/DSC data was used to find the thermal stabilities, glass transition temperatures, and enthalpies. Cyclic voltammetry measurements exhibited distinct oxidation and reduction peaks. NCs exhibited better potential as electrode materials for supercapacitor applications as compared to their respective NPs. NiO_et_@GO exhibited the best electrochemical performance and photocatalytic degradation efficiency of CV dye. After 120 min exposure to sunlight, the degradation coefficient of CV was observed to be 82.93, 86.34, 89.99, 90.27 and 81.65 % in the presence of NiO_aq_, NiO_et_, NiO_aq_@GO, NiO_et_@GO and GO, respectively.

## Highlights

• Synthesis of *E. cardamomum* mediated NiO and its nanocomposites with graphene oxide.
• Characterization was performed by XRD, SEM/EDX, FTIR, UV–Visible and TGA/DSC.
• Nanomaterials were found remarkable electrode materials for use in supercapacitors.
• NiOet@GO showed highest photocatalytic degradation efficiency of crystal violet dye.

## Graphical abstract

**Figure:**
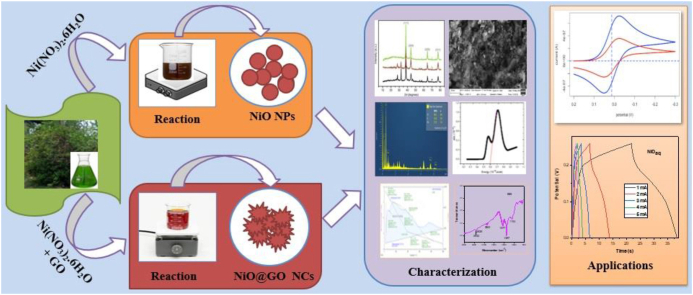

## 1 Introduction

In the last few years, nickel-based nanoparticles have attracted the attention of researchers due to their fantastic electronic, magnetic, catalytic, and optical properties and good stabilities [1]. They are widely applied in diverse fields, including fuel cells, gas sensors, memory storage devices, dye-sensitized solar cells, electrochromic devices, batteries, magnetic recording devices, and solar energy absorption [2,3]. Their remarkable efficiency in removing both inorganic and organic pollutants helps in environmental protection and pollution remediation e.g., by photocatalytic degradation of pollutants [4]. The nickel oxide (NiO) NPs find applications in semiconductors, batteries, thermistors and varistors, transparent heat mirrors, tuned circuits, capacitor–inductor devices, electrochromic and chemical or temperature sensing devices, micro-supercapacitors, active optical filters, aerospace, accelerators, radar absorbing materials, antiferromagnetic layers and in the preparation of textiles, plastics, numerous alloys, catalysts, nanofibers, nanowires and nickel cermet [5]. It is well established that the properties of metal nanoparticles can be improved by constructing their composites with carbon nanotubes [6] and graphene oxide [7].

The conventional physical/chemical approaches for the synthesis of NPs are associated with several drawbacks including high costs and the use/release of harmful toxic chemicals so there is a dire need to develop reliable, long-lasting and environmentally friendly synthetic protocols for NPs. Currently, the green synthetic route has been preferred due to its eco-friendly nature, environmental sustainability, cost-effectiveness [8] and reliable protocol and can be used to minimize the hazardous effects associated with traditional physical/chemical methods [9]. Since, this route is largely dependent on a variety of reaction parameters including biological precursors, nature of the solvent, temperature, pressure, pH conditions (acidic, basic, or neutral), the use of eco-friendly solvents (e.g., water, ethanol) and natural resources (such as plants) may play an important role in the development of such non-toxic protocols [10]. Green approaches using plant materials are highly beneficial for the manufacture of NPs since they are associated with myriads of health, economic, environmental and medicinal advantages [11]. The plant materials (especially leaves) due to their richness in numerous phytochemicals like ketones, aldehydes, flavones, amides, terpenoids, carboxylic acids, phenols and ascorbic acids [12], may serve as long-lasting and environmentally friendly reservoirs for the synthesis of metallic nanoparticles. The phytochemicals not only reduce the metal salt into metal/metal oxide NPs but they can also be adsorbed onto the surfaces of the NPs to provide them with stabilities [13].

Various green routes for the synthesis of NiO-based NPs were developed earlier. However, to the best of our knowledge, *E. cardamomum* mediated synthesis of nickel oxide (NiO) NPs and their composites with graphene oxide (GO), was never reported earlier. Current studies involve the synthesis of nickel oxide (NiO_aq_ and NiO_et_) NPs and their nanocomposites (NiO_aq_@GO and NiO_et_@GO) with GO by using aqueous and ethanolic extracts of *E. cardamomum* leaves as reducing and capping agents. The electrochemical properties of the bio-synthesized nanomaterials were investigated through cyclic voltammetry (CV) and galvanostatic charge-discharge (GCD) experiments. Their photocatalytic efficiency for the degradation of crystal violet (CV) dye was also evaluated. The investigated bio-synthetic route is easier, economical and eco-friendly and gives satisfactory yield. We have chosen the leaves of *E. cardamomum* for the nano-biosynthesis of nickel-based NMs because they are comprised of limonene, myrcene, sabinene, α-phellandrene, β-pinene, α-terpineol, citronellol, geranial, and trans-nerolidol [14] and thus, may act as natural reducing agents (to produce NMs) as well as stabilizing agents. Moreover, we have utilized water and ethanol solvents in our green synthetic routes because both these solvents are considered eco-friendly. Water is considered the best option solvent since it is the least expensive, safest and abundantly available everywhere. Moreover, it is non-flammable, non-toxic, affordable and has high boiling and critical points. Beyond its use in separating aqueous substances, water can widely be employed as both a medium and a reagent in various synthetic reactions [15]. Ethanol is also called a green solvent because it is a renewable source, which has negligible environmental and health issues and also occurs in nature. Ethanol, as a polar alcohol, can effectively solubilize polar/semi-polar compounds and can extract a diverse range of compounds, including flavonoids, alkaloids, and terpenes so it is widely employed in extraction processes. Ethanol also acts as a co-solvent with water, further expanding its solubilizing power [16].

## 2 Experimental

### 2.1 Materials and methods

Nickel nitrate hexahydrate, sodium nitrate (DAEJUNG company), ethanol (BDH laboratory suppliers), graphite (VWR chemicals Prolabo Company), hydrogen peroxide, hydrochloric acid, sulfuric acid, and potassium permanganate (SIGMA-ALDRICH) were used in research work. Beakers (100, 250 & 500 mL), hot plate (Italnee), Whatman filter paper, magnetic stirrer, spatula, Petri dish, thermometer, measuring cylinder, pipette, muffle furnace (Vulcan- D550), tripod stand, conical flask, funnel, dropper, and Pyrex origin glassware were also used.

The phase nature and crystallinity of the synthesized nanomaterials were examined by the X'Pert Powder X-Ray Diffractometer. FT-IR spectroscopy was performed by FT-IR-8400 spectrometer in the range of 400–4000 cm^−1^. The morphology of the synthesized nanomaterials was examined by using SEM analysis (Zeiss Sigma 500 VP). EDX analyzer (Zeiss Sigma 500 VP) was used to determine the elemental composition of NPs and NCs. UV–visible spectroscopy was performed by using a spectrometer (BIOBASE BK-D560) in the range of 200–800 nm. The thermal analysis (TGA-DSC) was conducted utilizing a Discovery 650 SDT thermal analyzer from TA Instruments, USA, over a temperature range of 25–1000 °C. The heating rate employed was 20 °C per minute, and ultra-pure N_2_ (50 mL/min) was used as the inert gas. The electrochemical behavior of the synthesized NMs was investigated by using a Galvanostat/Potentiostat of the CS300 model. We also tested the photocatalytic degradation of crystal violet (CV) dye in the presence of synthesized nanomaterials whereas two-way ANOVA was applied for statistical analysis of the obtained results.

### 2.2 Collection of Elettaria cardamomum leaves and extract formation

The investigated plant of *E. cardamomum* (Fig. S1, Supplementary Information) was identified by the Department of Life Sciences, KFUEIT Rahim Yar Khan (Punjab, Pakistan). The plant leaves were collected from a village of Sehja, district Rahim Yar Khan (Punjab, Pakistan) on August 12, 2022, washed with clean tap water and dried under shade. The leaves were converted into a fine powder by grinding them in a kitchen blender.

The aqueous extract of *E. cardamomum* leaves was prepared by mixing 10g of leaves powder with 150 mL of distilled water. The mixture was continuously stirred at a temperature of 90 °C for 2 h in a beaker covered with aluminum foil. The contents of the beaker were then cooled and filtered by using Whatman filter paper to leave behind the aqueous extract (filtrate), which was stored at 4 °C.

The same procedure was adopted to produce ethanolic extract of *E. cardamomum* by stirring the plant leaves powder (10 g) with ethanol (150 mL) for 2 h at room temperature, followed by cooling and filtration.

### 2.3 Preparation of nanoparticles (NiO_aq_ and NiO_et_)

100 mL aqueous extract of *E. cardamomum* leaves was stirred with 100 mL of 0.1 mM nickel nitrate hexahydrate solution at 80 °C for 3 h. The reaction contents were stayed overnight at room temperature to settle down the precipitates which were collected by centrifugation and dried in the oven at 70 °C. The dried precipitates (0.01 g) were calcined at 400 °C for 2 h to produce the NiO_aq_ NPs (0.007 g, final product).

The same procedure was used to form NiO_et_ by stirring *E. cardamomum* leaf extract (100 mL) and 0.1 mM nickel nitrate hexahydrate solution (100 mL) for 3 h at room temperature. The dried precipitates (0.008 g) were calcined at 400 °C for 2 h to produce the final product of NiO_et_ NPs (0.006 g).

The whole green synthetic route for the formation of NiO_aq_ and NiO_et_ NPs using aqueous/ethanolic extracts of *E. cardamomum* leaves has been displayed in Fig. S2 (Supplementary Information).

### 2.4 Synthesis of graphene oxide (GO)

GO was prepared using the Hummers' method [17]. Within an ice bath, a mixture of 200 mL of H_2_SO_4_ and 0.5 g of graphite powder was stirred mechanically in a 500 mL beaker. The temperature of the suspension was maintained below 5 °C. Then, 5 g of KMnO_4_ and 8 g of NaNO_3_ were slowly added to the beaker. The mixture was continuously agitated for four days. After this period, 100 mL of distilled water was added, and the solution was allowed to cool down. Next, 6 mL of 30 % H_2_O_2_ was added, and the mixture was subjected to heating at 90 °C for 1 h. Subsequently, the solution was left for a day to allow the precipitates to settle down. The solution was then subjected to centrifugation and washed with a 0.1 % aqueous solution of HCl to eliminate the metal ions. Rinsing was done with distilled water till all the unreacted acid was removed. Finally, the solution was freeze-dried to obtain GO. The synthetic route for the formation of GO has been displayed in Fig. S3 of Supplementary Information.

### 2.5 Formation of composites (NiO_aq_@GO & NiO_et_@GO)

A suspension of 20 mg of GO in 15 mL distilled water was added to 5 mL nickel nitrate hexahydrate solution (0.1 M), and the mixture was stirred for 1 h. Then 5 mL aqueous extract of *E. cardamomum* leaves was added and the stirring was continued at 80 °C for an hour till there was a slight color change. The solution was subjected to centrifugation at 4000 rpm; the obtained precipitates of NiO_aq_@GO NCs were dried in an oven at 45 °C and finally stored for further analysis and use. The same process was used to prepare NiO_et_@GO NCs at room temperature by using the ethanolic extract of *E. cardamomum* leaves in place of their aqueous extract. 0.1 M nickel nitrate hexahydrate solution was used for the preparation of nanocomposites because, at this concentration, the yield of the composites was optimum. Fig. S4 of Supplementary Information shows the *E. cardamomum* mediated synthetic route of NiO_aq_@GO and NiO_et_@GO nanocomposites.

### 2.6 Electrochemical investigations

The electrochemical behavior of the prepared NPs (NiO_aq_ & NiO_et_) and their NCs (NiO_aq_@GO & NiO_et_@GO) was investigated by using a reported procedure for galvanostatic charging discharging (GCD) and cyclic voltammetry (CV) [18]. A three-electrode (working, counter & reference) system was utilized for the electrochemical investigations. The NMs (NiO_aq_, NiO_et_, NiO_aq_@GO, NiO_et_@GO and GO) were used as working electrodes and deposited onto the nickel foam. The Ag/AgCl system and a Pt wire were used as reference and counter electrodes, respectively, with 6M KOH solution as an electrolyte. The electrochemical material had a loading density of 0.1 mg cm^−2^. KOH was chosen as the electrolyte material due to its excellent conductivity. Additionally, its 6M concentration yields an excellent concentration of OH^−^ ions, facilitating charge transfer in the bulk electrodes. A tiny piece of Ni foam was used to make the electrode material, which was then sonicated for 10 min firstly with ethanol and then with deionized water after being cleaned with the same solvents. The nickel foam was then dried using a drier. Then a mixture containing 80 % NMs, 15 % carbon charcoal and 5 % binder was ground into a fine powder in a pestle and mortar, followed by the addition of a few drops of NMP to create a slurry which was finally applied on the nickel foam and dried at 70 °C [18].

### 2.7 Photocatalytic degradation of NMs

The photocatalytic efficiency of NMs depends on the degradation ability of a dye in the presence of photocatalysts. We have used sunlight as UV–visible source. A stock solution of crystal violet (CV) dye was prepared by mixing 0.003 g of CV in 100 mL water followed by stirring for 10 min. Then, 20 mg of a synthesized nanomaterial (NiO_aq_, NiO_et_, NiO_aq_@GO, NiO_et_@GO and GO) was separately added into a 50 mL solution of CV and the mixture was continuously stirred for 30 min in the dark. Finally, 3 mL of the suspension was collected from the reaction mixture and its absorbance was measured by a UV–visible spectrophotometer; the remaining solution was exposed to sunlight. Then, 3 mL suspension was collected periodically from the sun-irradiated mixture at different time intervals of 30, 60, 90 and 120 min and its absorbance was measured in the same way. The rate of dye degradation of a dye sample collected for each time (3 mL) was calculated from its measured value of the absorbance.

## 3 Results and discussions

Aqueous and ethanolic extracts of *E. cardamomum* leaves were treated with nickel nitrate hexahydrate to produce NiO_aq_ and NiO_et_ NPs, respectively; the same reaction in the presence of graphene oxide (GO) resulted in the formation of NiO_aq_@GO and NiO_et_@GO nanocomposites. The NMs were characterized by XRD, SEM, EDX, FT-IR, UV–visible spectroscopy, and TGA-DSC analysis. The electrochemical applications were performed by using cyclic voltammetry and charging-discharging behavior evaluation. The photocatalytic efficiency of MMs was tested by the degradation of crystal violet (CV) dye in the presence of sunlight.

### 3.1 X-ray diffraction (XRD) analysis

**Fig. 1:**
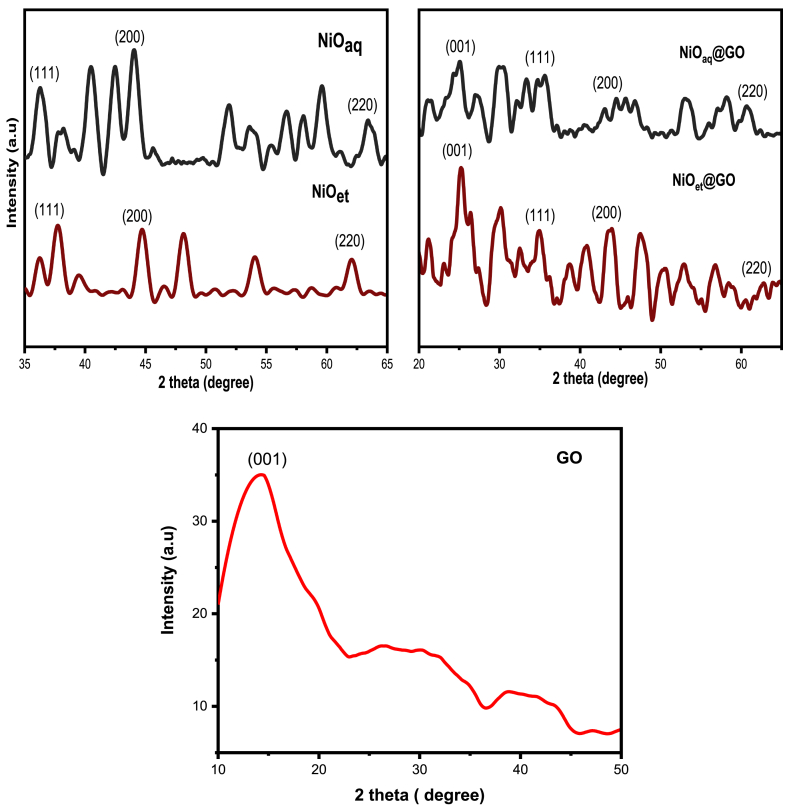
XRD spectra of NiO_aq_, NiO_et_, NiO_aq_@GO, and NiO_et_@GO and GO.

The phase nature and crystallite sizes of synthesized NMs were found by analyzing their X-ray diffraction patterns. The XRD graphs of NiO_aq_, NiO_et_, NiO_aq_@GO, NiO_et_@GO and GO are shown in Fig. 1.

The diffraction peaks of NiO_aq_ at 36.32°, 44.05°, and 63.50° correspond to the (111), (200), and (220) planes, respectively, and these peaks indicate a cubic structure. The XRD spectrum of NiO_et_ displayed distinctive peaks at 2θ angles of 37.73°, 44.72°, and 62.05°, which corresponded to various diffracting planes of cubic NiO crystallites, namely (111), (200), and (220), respectively. In NiO_aq_@GO the diffraction peaks located at 2θ values of 35.32°, 44.86° and 60.55° correspond to the crystal planes of (111), (200), and (220), respectively. The diffraction peaks of NiO_et_@GO were located at 2θ values of 38.69°, 43.71° and 62.67° corresponding to the crystal planes of (111), (200), and (220), respectively [19]. However, in both NiO_aq_@GO and NiO_et_@GO composites, an additional peak was shown at about 25.01° and 25.23°, respectively. This peak corresponds to the (001) crystal plane of the graphene oxide (GO) sheets [20]. The NiO NPs, NiO@GO NCs and GO have JCPDS Cards 47–1049, 00-012-0771 and 01-075-1621, respectively. The XRD pattern of sample GO (Fig. 1) showed a peak at 13.8° which corresponds to the (001) plane [21]. However, in NiO@GO NCs, the interaction between GO and the NiO can further influence the reduction process and the resulting structure. These interactions can promote the removal of oxygen groups and improve the stacking order of the graphene layers. Therefore, the peak of GO was significantly shifted to 25.01° and 25.23° (001 crystal plane) when it interacted with NiO NPs in NiO_et_@GO and NiO_et_@GO NCs, respectively [22].

The size of crystallite species can be calculated by using the Debye–Scherrer formula (Eq. (1)).

(Eq. 1) $$D=\frac{k\lambda }{\beta \;\cos\;\theta }$$

Where K = 0.94 which is a crystallized form factor, β represents the peak's highest width at half maximum, λ is the wavelength (0.154), D is particle size and θ is the diffraction angle. The sizes of NiO_aq_, NiO_et_, NiO_aq_@GO, and NiO_et_@GO were found to be 14.07, 12.15, 9.15, and 8.84 nm, respectively. The presence of GO can inhibit the crystal growth of NiO due to spatial confinement effects. The layered structure of GO can physically restrict the growth of NiO crystallites, resulting in smaller sizes. When GO controls the sizes of NPs, in applications like batteries and supercapacitors, smaller crystallite sizes can improve performance by providing more active sites for electrochemical reactions [23]. The combination of NiO and GO enhances the conductivity and stability of the electrode material, leading to better charge-discharge cycles and limiting the long-range order of crystal growth [24]. In our studies, NiO_et_@GO has shown the lowest crystallite size (8.84 nm) so theoretically, it is expected more active material. These findings are in good agreement with the results of our investigated products where NiO_et_@GO has shown better electrochemical performance (Section 3.7) as well as photocatalytic degradation of crystal violet dye (Section 3.8), as compared to the remaining biosynthesized materials.

### 3.2 FT-IR spectroscopy

**Fig. 2:**
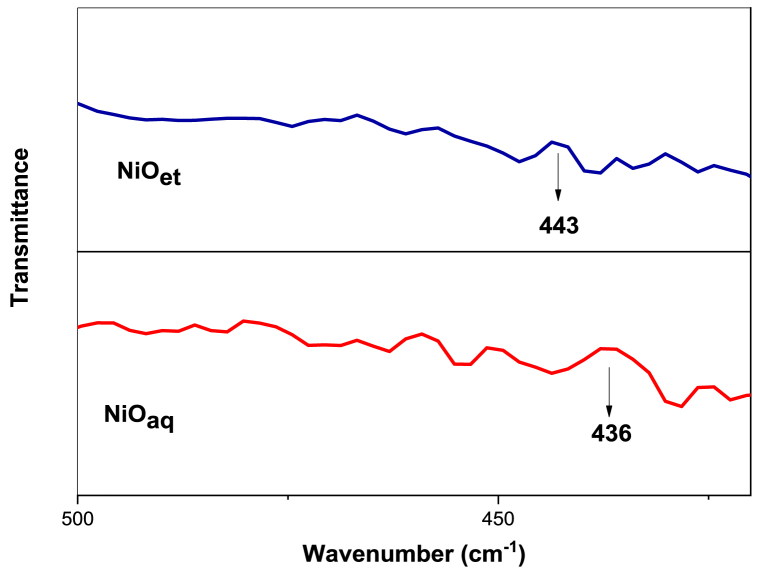
FT-IR stacked spectrum of NiO_aq_ and NiO_et_.

**Fig. 3:**
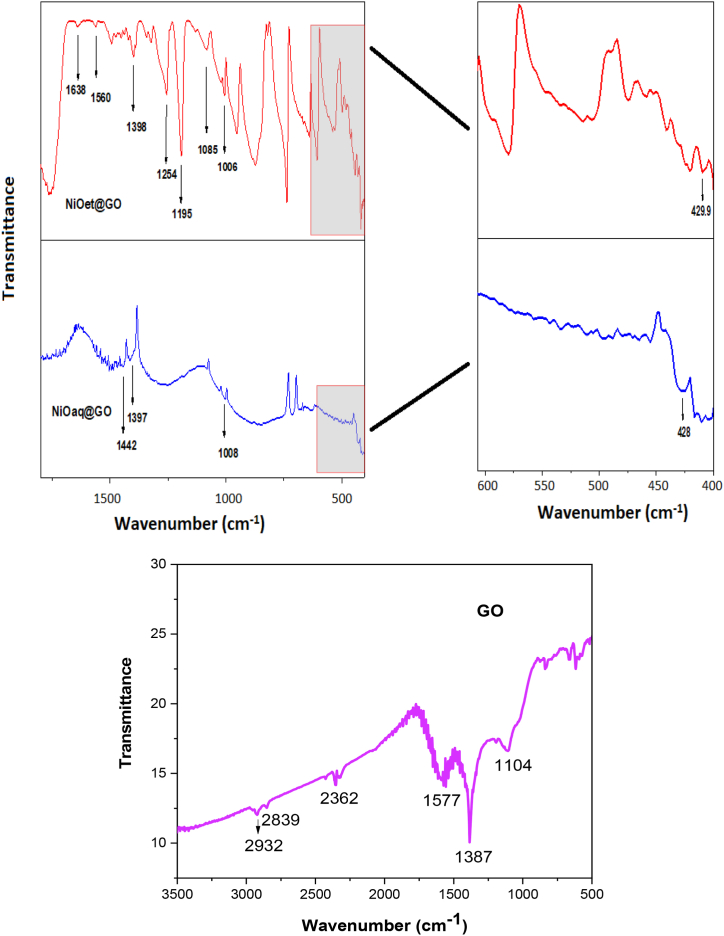
FT-IR spectra of NiO_aq_@GO and NiO_et_@GO and GO.

FTIR-spectroscopy is a very useful technique for the structural interpretation of nanomaterials [25]. The FT-IR spectra were recorded for our synthesized NMs in a range of 400–4000 cm^−1^ and are disclosed in Fig. 2, Fig. 3.

Ni-O vibrations appeared at 443 and 436 cm^−1^ in the FTIR spectra of NiO_aq_ and NiO_et_, respectively [26]; they were shifted to 428 and 429.9 cm^−1^ in the respective nanocomposites i.e., NiO_aq_@GO and NiO_et_@GO, respectively. The incorporation of GO into the NiO structure can lead to significant interactions between the NiO NPs and the functional groups present on the GO sheets. These interactions can alter the local environment of the Ni-O bonds, leading to a shift in their vibrational frequencies. NiO_et_@GO showed bands for the C=O stretching mode of ketonic species (1638 cm^−1^) [27], aromatic C=C (1560 cm^−1^) [28], C-O symmetric stretching vibration of epoxy (1254 cm^−1^) [29,30], C-O-C stretching (1195 cm^−1^) [31] and C-OH bending vibrations (1085 cm^−1^) [30]. The unoxidized graphitic domains (C-O-C vibrations) were observed at 1442 cm^−1^ in NiO_aq_@GO [29]. The stretching vibrations of a phenolic hydroxyl group (C-OH) were observed at 1397 and 1398 cm^−1^ in NiO_aq_@GO and NiO_et_@GO, respectively. The peaks at 1006 and 1008 cm^−1^ were assigned to C-O-C stretching vibrations in NiO_et_@GO and NiO_aq_@GO NCs, respectively [31].

Graphene oxide (GO) showed bending vibrations of C-H bonds (1104 cm^−1^), C-OH stretching vibrations of the phenolic hydroxyl group (1387 cm^−1^) [31], and aromatic C=C group (1577 cm^−1^) [28]. The appearance of two absorption bands at 2362 and 2839 cm^−1^ indicates the successful splicing of O_2_-based functional groups on the surface and edges of the graphite sheet and corresponds to the stretching vibrations of C-H bonds [32]. The peak at 2932 cm^−1^ provides evidence for the existence of asymmetric and symmetric stretching vibrations of CH_2_ bonds [29].

### 3.3 SEM analysis

**Fig. 4:**
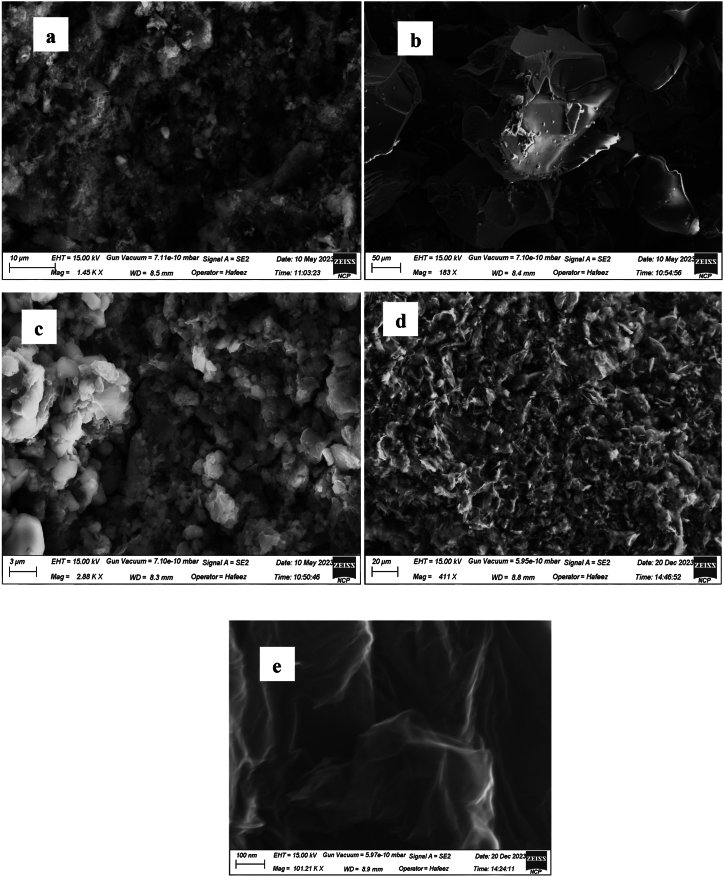
SEM images of NiO_aq_ (a), NiO_et_ (b), NiO_aq_@GO (c), NiO_et_@GO (d) and GO (e).

**Fig. 5:**
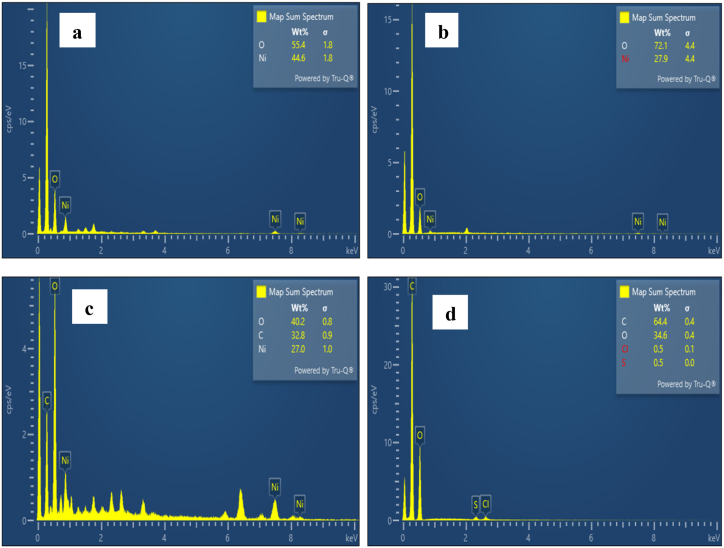
EDX spectra of NiO_aq_ (a), NiO_et_ (b), NiO_aq_@GO (c) and GO (d).

Fig. 5 illustrates the morphology of the synthesized NiO_aq_, NiO_et_, NiO_aq_@GO, NiO_et_@GO and GO nanomaterials. NiO_aq_ NPs (Fig. 4a) exhibited significant particle agglomeration. Additionally, the image displays smaller grains dispersed randomly, suggesting the presence of homogeneous spherical-shaped particles. The particle size, as depicted in the image (Fig. 4a), is measured to be 30.63 nm for NiO_aq_ NPs. NiO_et_ NPs, exhibited spherical particles, which are significantly agglomerated, appearing as clusters (Fig. 4b). The particle size of NiO_et_ NPs was found to be 27.7 nm.

NiO_aq_@GO was depicted as having randomly aggregated graphene sheets (Fig. 4c), that are thin and exhibit wrinkles; their particle size was 26.39 nm. The SEM image of NiO_et_@GO (Fig. 4d) shows that particles have chaotic structure, wrinkled graphene sheets, thin, and randomly aggregated, close to each other. The particle size of NiO_et_@GO NC was found to be 19.33 nm. The GO has thin layers with curved and wrinkled structures having a particle size of 43.33 nm (Fig. 4e).

### 3.4 EDX analysis

**Fig. 6:**
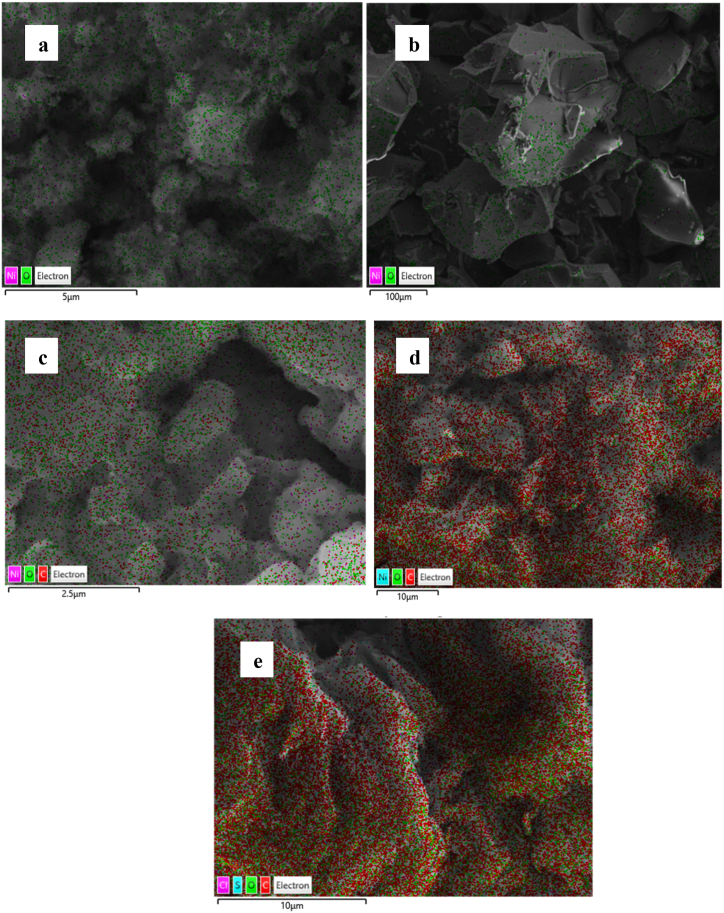
Elemental mapping of NiO_aq_ (a), NiO_et_ (b), NiO_aq_@GO (c), NiO_et_@GO (d) and GO (e).

The EDX spectra of NiO_aq_ and NiO_et_ NPs showed the presence of Ni and O (Fig. 5a–b); the EDX spectrum of NiO_aq_@GO shows the presence of C, O, and Ni (Fig. 5c); the EDX spectrum of GO shows the presence of C and O (Fig. 5d). Weight percentages reveal distinct peaks of Ni: 44.6 % for NiO_aq_, 27.9 % for NiO_et_, and 27.0 % for NiO_aq_@GO. Similarly, O shows clear peaks: 55.4 % in NiO_aq_, 72.1 % in NiO_et_ NPs, 40.2 % in NiO_aq_@GO and 34.6 % in GO. The weight percentage of carbon in NiO_aq_@GO and GO was observed to be 32.8 and 64.4 %, respectively. The atomic percentages of the elements are shown in Table 1. The mapping result of NMs (Fig. 6) demonstrates that elements were homogeneously distributed on the surfaces of the particles.

**Table 1:** Atomic weight percentage of nanoparticles.

Elements	NiO_aq %_	NiO_et %_	NiO_aq_@GO %	GO %
Ni	20.4	9.62	8.07	–
O	79.6	90.38	44.04	28.9
C	–	–	47.89	71.1

### 3.5 UV–visible analysis

**Fig. 7:**
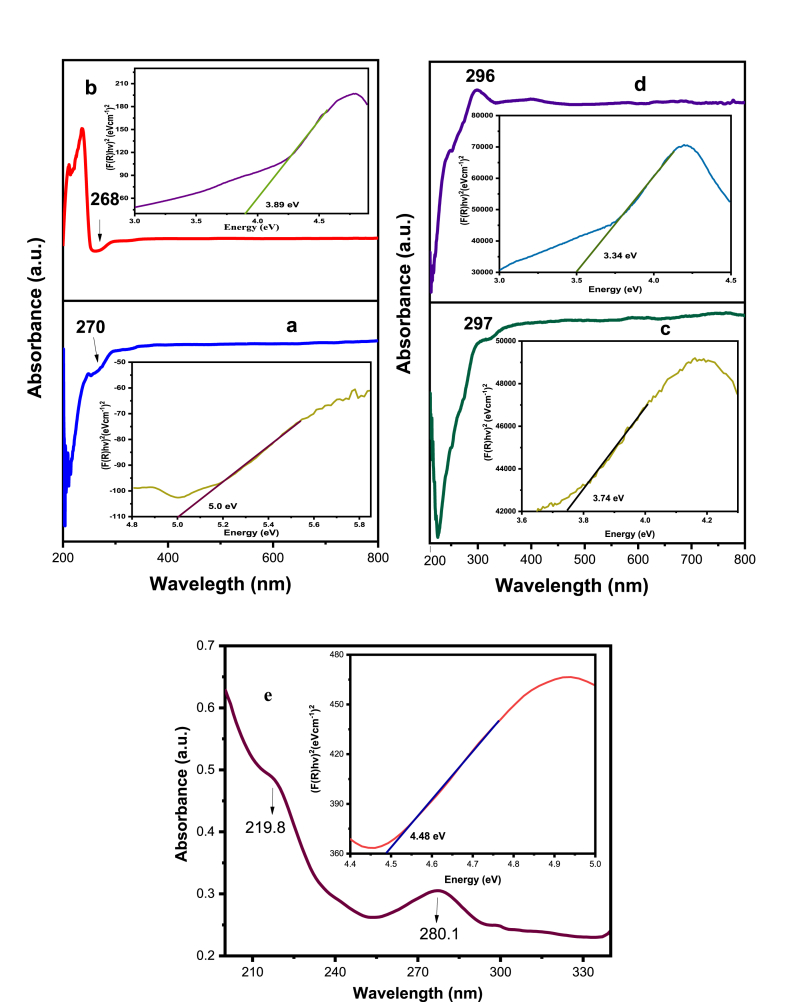
UV–visible spectra with band gaps of NiO_aq_ (a), NiO_et_ (b), NiO_aq_@GO (c), NiO_et_@GO (d) and GO (e).

Fig. 7a-e demonstrates the UV–visible spectra of the NiO_aq_ and NiO_et_ NPs, their NCs (NiO_aq_@GO and NiO_et_@GO) and GO. An absorption peak was observed at a wavelength of 270 and 268 nm in NiO_aq_ and NiO_et_ NPs, respectively (Fig. 7a–b); this peak was shifted to 297 and 296 nm in NiO_aq_@GO and NiO_et_@GO, respectively due to the confinement of charge carriers in NiO NPs (Fig. 7c–d) [33]. The graphene oxide exhibited a first band at 219.8 nm and a second band at 280.1 nm (Fig. 7e); these spectral features correspond to the π–π∗ transitions of C=C bonds and the n–π∗ transition of C=O bonds, respectively [34]. The band gaps of NiO_aq_, NiO_et_, NiO_aq_@GO, NiO_et_@GO, and GO were calculated by using the Kubelka–Munk function plot expression (Eq. (2)).

(Eq. 2) $$F\left(R\right)=\frac{{\left(1-R\right)}^{2}}{2R}$$

Where R is the reflectance value. The bandgap energy of each sample was estimated from the variation of the Kubelka-Munk function with photon energy [35]. The band gaps of NiO_aq_, NiO_et_, NiO_aq_@GO, NiO_et_@GO, and GO were found to be 5.0, 3.89, 3.74, 3.34, and 4.48 eV, respectively (Fig. 7a–e). Due to the incorporation of GO, the synthesized NCs have shown smaller band gaps (NiO_aq_@GO = 3.74 eV; NiO_et_@GO = 3.34 eV) as compared to their respective NPs (NiO_aq_ = 5.0 eV; NiO_et_ = 3.89 eV). The redshifts in bandgap values of NCs (NiO_aq_@GO and NiO_et_@GO) as compared to their respective NPs (NiO_aq_ and NiO_et_) suggest the formation of additional energy levels within the valence band (VB) and conduction band (CB) of NiO NPs due to the inclusion of graphene oxide. This modification enhances the emission behavior in the visible region [36].

### 3.6 Thermogravimetric (TGA-DSC) analysis

**Fig. 8:**
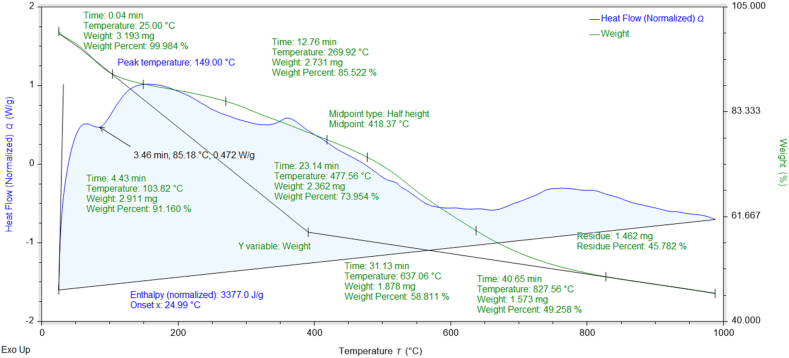
TGA-DSC curves of NiO_aq_ NPs.

**Fig. 9:**
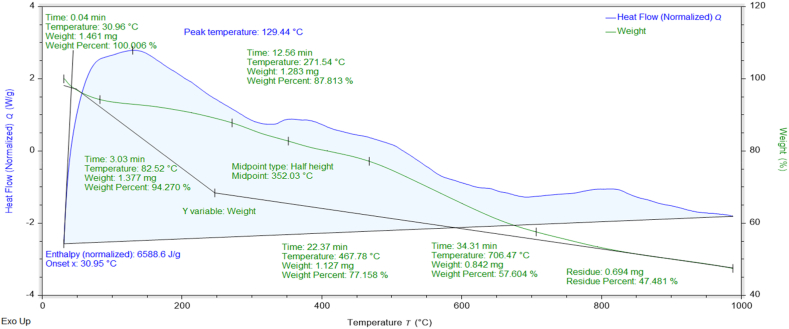
TGA-DSC curves of NiO_et_ NPs.

**Fig. 10:**
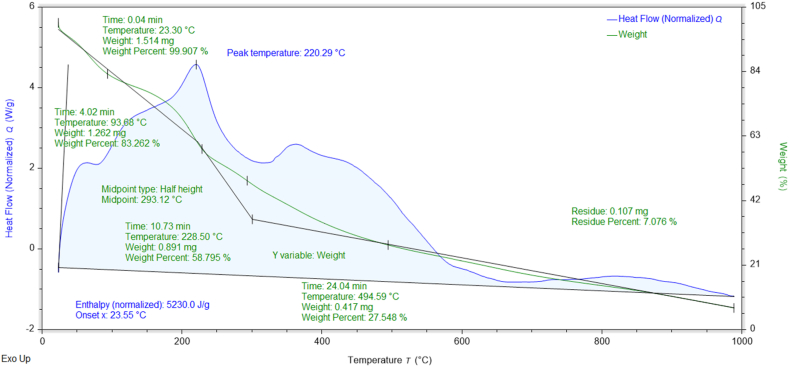
TGA-DSC curves of NiO_aq_@GO

**Fig. 11:**
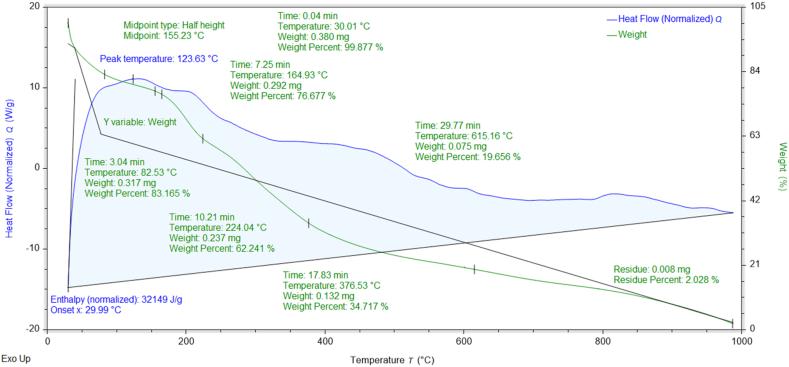
TGA-DSC curves of NiO_et_@GO

**Fig. 12:**
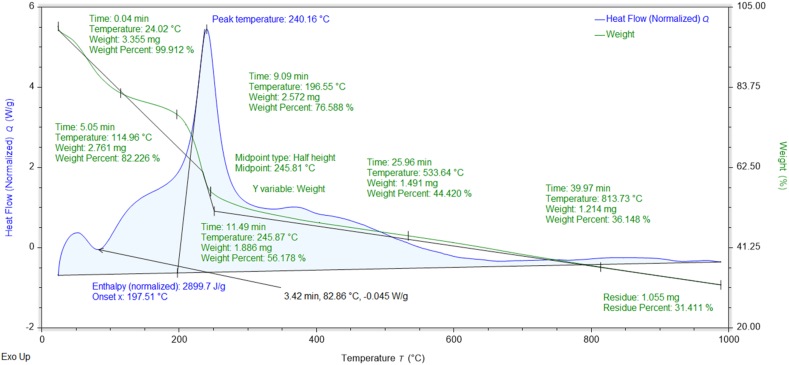
TGA-DSC curves of GO.

The percent weight losses of NMs concerning temperature and their thermal stabilities can be investigated through TGA data [37]. The thermograms and DSC curves of the synthesized nanomaterials are shown in Fig. 8, Fig. 9, Fig. 10, Fig. 11, Fig. 12. The total weight losses of NiO_aq_, NiO_et_, NiO_aq_@GO, NiO_et_@GO, and GO were found to be 54.22, 52.52, 92.92, 97.9, and 68.6 %, respectively. The TGA graphs (Fig. 8, Fig. 9) revealed a three-staged thermal degradation pattern for the weight loss and decomposition of NiO_aq_ and NiO_et_. The initial weight loss at 25–100 °C can be attributed to the loss of OH^−^ groups or evaporation of water molecules (moisture) from NiO_aq_ and NiO_et_. A sudden second weight loss occurred between 268 and 478 °C, was primarily attributed to the decomposition of Ni(OH)_2_ to NiO NPs. Simultaneously, a continuous third weight loss was observed in the 635–830 °C range, indicative of NiO NPs formation [38]. The DSC curves showed the endothermic peaks in NiO_aq_ and NiO_et_ at 83 °C and 280 °C. The exothermic peaks were seen at 360 °C in DSC curves of NiO_aq_ and at 362 & 812 °C in NiO_et_.

The NiO_aq_@GO and NiO_et_@GO NCs (Fig. 10, Fig. 11) have shown a small weight loss up to 100 °C. However, a major weight loss was observed in the NCs from 220 to 445 °C, which can be attributed to the loss of oxygen functional groups present on the GO sheets. In DSC curves, an endothermic peak was observed at 78 °C in NiO_aq_@GO and at 172 °C in NiO_et_@GO. The exothermic peaks were shown at 220 and 360 °C in NiO_aq_@GO.

In the case of GO nanosheets (Fig. 12), three well-defined weight loss patterns can be identified at three different temperature regions: the first stage occurring between 24 and 114 °C is ascribed to the evaporation of the trapped residual water on the surfaces of GO sheets; the second weight loss observed in the range of 196–246 °C corresponds to the elimination of the oxygen-based functional groups (i.e. hydroxyl, carboxylic and epoxide groups) from the surface and the edges of GO nanosheets, which are removed in the form of gases such as H_2_O and CO_2_ as byproducts [39]. The concluding stage, occurring in the temperature range of 513–815 °C, can be linked to the oxidative pyrolysis of the carbon framework.

TGA-DSC; Enthalpy (normalized): 3377.0, 6588.6, 5230, 32149, and 2899.7 J/g in NiO_aq_, NiO_et_, NiO_aq_@GO, NiO_et_@GO, and GO, respectively; Glass transition temperature at midpoint of half height: 418.3, 352.03, 293.12, 155.23, and 245.81 °C in NiO_aq_, NiO_et_, NiO_aq_@GO, NiO_et_@GO, and GO, respectively.

### 3.7 Electrochemical investigations

#### 3.7.1 Cyclic voltammetry

**Fig. 13:**
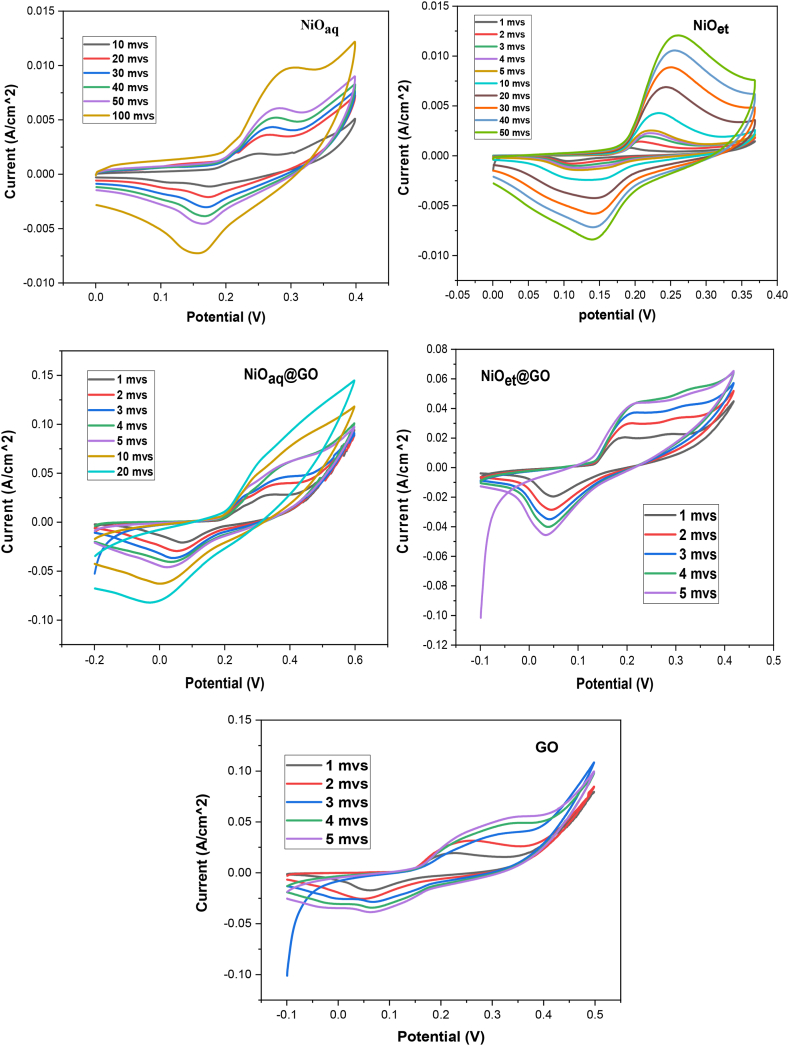
Cyclic voltammograms of NiO_aq,_ NiO_et_, NiO_aq_@GO, NiO_et_@GO and GO.

The CV curves of NiO NPs (NiO_aq_ and NiO_et_) and their NCs (NiO_aq_@GO and NiO_et_@GO) (Fig. 13) exhibited prominent oxidation and reduction peaks in the potential range of −0.5 to +0.5 V. The transfer of electrons and ions makes these oxidation and reduction reactions kinetically reversible. In case of kinetic irreversibility of the oxidation and reduction reactions, during the cathodic and anodic scans, asymmetrical reduction and oxidation peaks should be observed.

On the cyclic voltammograms, the redox behaviors of NMs and GO were confirmed through the observation of distinct oxidation-reduction peaks. The CV graphs displayed slight variations in the reduction peak potential ranging from 0.1 to 0.2 V in NiO_aq_/NiO_et_ NPs and from −0.1 to 0.1 V in NiO_aq_@GO, NiO_et_@GO, and GO. Furthermore, it was noted that the oxidation peaks showed similar patterns in the range of 0.2–0.32 V in NMs and GO. Notably, all the CV curves of the NMs exhibited redox peaks, providing evidence that the charging processes of all synthesized NiO NPs and their NCs are reversible. The NCs demonstrate a higher maximum current and a reduced peak-to-peak separation potential, illustrating the synergistic effect of NiO and GO nanosheets on enhancing conductivity and accelerating charge transfer [18]. This combination results in increased catalytic activity and improved accessibility to catalytic active sites. Due to its extensive surface area and enhanced porosity, NCs exhibited amplified peak current and diminished peak potential [40].

#### 3.7.2 Galvanostatic charging discharging behavior

**Fig. 14:**
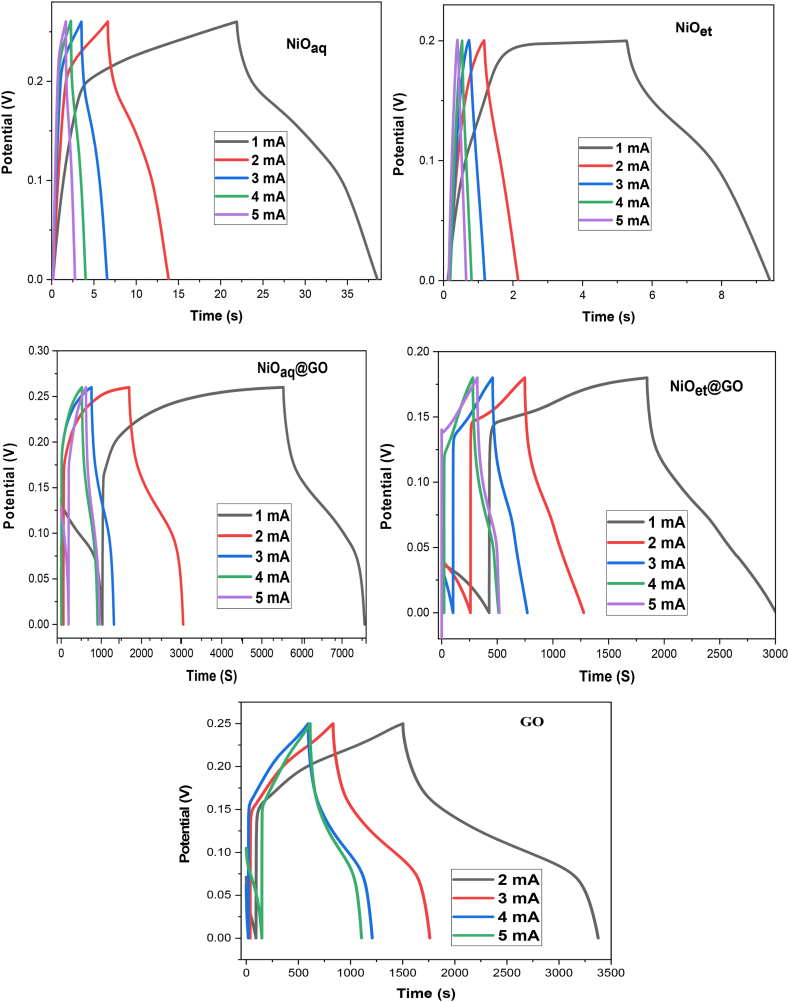
Galvanostatic charge and discharge of NiO_aq_, NiO_et_, NiO_aq_@GO, NiO_et_@GO and GO.

At various current densities, we investigated the galvanostatic charge-discharge behavior of synthesized NMs. The good charge-discharge properties were shown even at higher current densities. The obtained charge-discharge profiles are depicted in Fig. 14, which were carried out in the potential range of 0–0.35 V. A nonlinear curve in the charging process between 0 and 0.35 V suggests that an electrochemical reaction has taken place. The nonlinear charge-release profile exhibits the pseudo-capacitive behavior of the NiO NPs [1].

The specific capacitance of NiO NPs and their NCs was determined by using Equation (3).

(Eq. 3) Cs=IΔt/mΔV

Where V is the potential difference, I is the constant current (A), t is the discharging time (s), and m is the electrode's mass (g) [1]. Accordingly, results were concluded for nanoparticles and their composites by utilizing GCD charts given in Table 2. The obtained results show that the specific capacitance, energy density, and power density of the NCs were higher as compared to NiO NPs. Therefore, GO-based electrodes could serve as potential supercapacitor electrodes due to their impressive performance [41].

**Table 2:** Specific capacitance and energy density of investigated NPs and their NCs.

Sample name	Current density	Specific capacitance	Energy density	Power density
NiO_aq_	1	61.07	2.09	0.13
NiO_et_	1	20	0.4	0.1
NiO_aq_@GO	1	7581.4	716.4	0.35
NiO_et_@GO	1	6005.26	108.3	0.094
NiO_aq_	2	65.45	1.58	0.2
NiO_et_	2	9.23	0.18	0.2
NiO_aq_@GO	2	10091.69	541.09	0.25
NiO_et_@GO	2	5623.88	91.10	0.17
GO	2	14604.16	420.5	0,28
NiO_aq_	3	33	1.11	0.39
NiO_et_	3	6.6	0.13	0.3
NiO_aq_@GO	3	6376.84	215.53	0.83
NiO_et_@GO	3	5108.33	82.75	0.26
GO	3	11059.24	319.53	0.028
NiO_aq_	4	26.46	0.89	0.51
NiO_et_	4	4.05	0.08	0.3
NiO_aq_@GO	4	5760.30	194.69	0.5
NiO_et_@GO	4	5070	82.13	0.35
GO	4	10064.5	289.8	0.028
NiO_aq_	5	76.15	2.57	0.64
NiO_et_	5	3.6	0.07	0.3
NiO_aq_@GO	5	6548.07	221.32	0.64
NiO_et_@GO	5	5413.05	87.69	0.44
GO	5	10066.6	289.91	0.028

The galvanostatic charging discharging curves also clarify that the charging and discharging times of the NCs are greater than that of the nanoparticles (Table 3) [1]. Additionally, graphene oxide can inhibit the accumulation of NiO NPs throughout the process of charging/discharging, whereas the presence of NiO NPs can impede the collection of graphene oxide [42]. These findings demonstrate that NCs (NiO_aq_@GO and NiO_et_@GO) are superior materials for energy storage applications compared to NPs (NiO_aq_ and NiO_et_) [1]. It is due to the low crystallite sizes and high surface areas of NiO_aq_@GO and NiO_et_@GO as compared to NPs (NiO_aq_ and NiO_et_) as discussed in Section 3.1.

**Table 3:** Charging and discharging capacity of NiO NPs and their NiO@GO NCs.

Sr. No	Sample name	Charging time	Discharging time
1	NiO_aq_	13.16668	18.66
2	NiO_et_	3.266675	5.544433
3	NiO_aq_@GO	1059.578	2258.2918
4	NiO_et_@GO	1216.547	1552.713
5	GO	1105.83	1540.46

### 3.8 Photodegradation performance

**Fig. 15:**
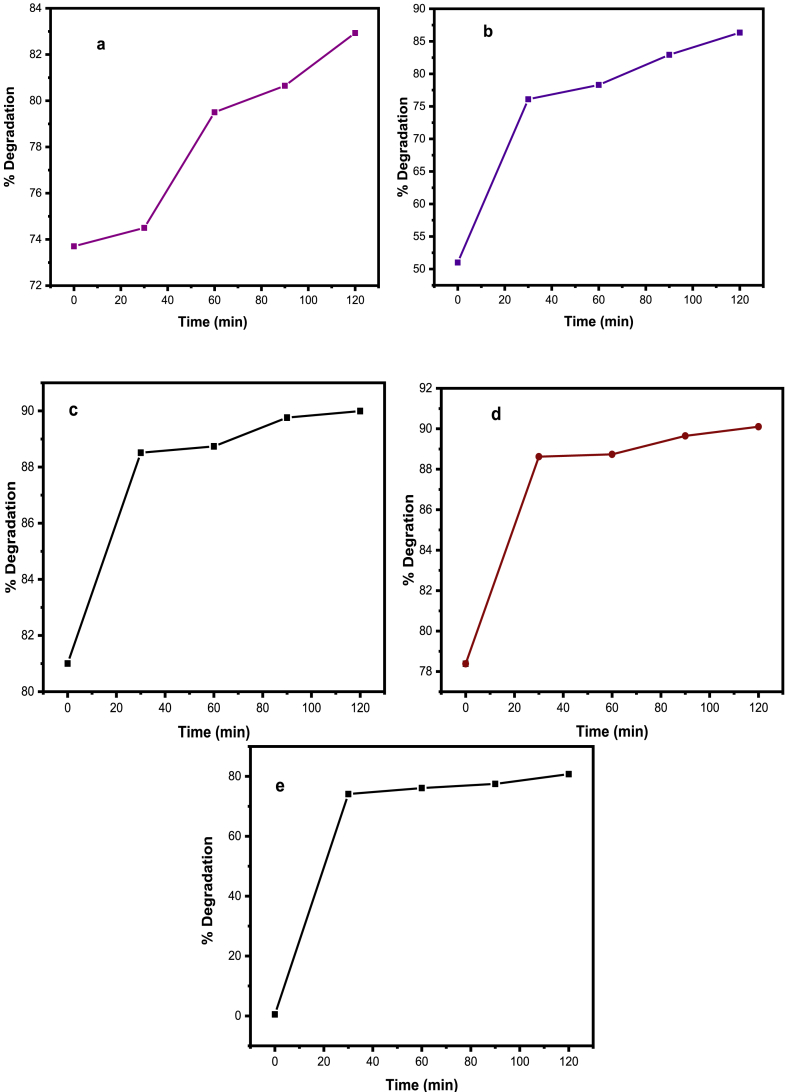
Photodegradation efficiency of CV in the presence of NiO_aq_ (a), NiO_et_ (b), NiO_aq_@GO (c), NiO_et_@GO (d) and GO (e).

The photocatalytic activity of NMs was determined by testing the photodegradation of CV solution under sunlight irradiation. The UV–visible spectra (Fig. S5, Supplementary Information) demonstrate the maximum absorbance of CV at a wavelength of 580–583 nm. The NiOet@GO photocatalyst showed the best photocatalytic degradation (90.27 %) of CV dye in its aqueous solution at an irradiation time of 120 min whereas the remaining nanomaterials i.e., NiO_aq_, NiO_et_, NiO_aq_@GO, NiO_et_@GO and GO exhibited 82.93, 86.34, 89.99 and 81.65 % dye degradation, respectively in the same timing (120 min). Fig. 15 illustrates the percentage photodegradation of CV over irradiation time. The degradation of CV dye was increased as the irradiation time progressed and was calculated by using the following formula (Eq. (4)).

(Eq. 4) $$\text{Degradation}\;\left(\%\right)=\left(A_{0}-A_{t}\right)/A_{0}\times 100\%$$

Where A_0_ and A_t_ represent the absorbance values of the dye before and after the photodegradation tests, respectively. The high reaction rate is attributed to the small size of the biosynthesized NiO@GO nanocomposites, which allows more surface atoms to participate in the reaction compared to bulk materials. This is due to the large surface-to-volume ratio, which enhances the number of reactant molecules that can interact and react simultaneously [43].

The kinetic behavior of the photocatalytic degradation process was also investigated using Eq. (5)) [44].

(Eq. 5) $${\text{InC}}_{t}/C_{o\;}=\left(K_{\text{obs}}\right)t$$

Here, C_o_ represents the initial concentration of CV dye at time t = zero while C_t_ denotes the concentration at variable time t and k_obs_(1/min) is the first-order rate constant. Fig. S6 of Supplementary Information represents the Langmuir isotherm model plot of InC_t_/C_o_ versus time under UV and sunlight irradiation. The results showed that linear relationships with good correlations are obtained, which indicates that the reactions follow pseudo-first order. It is also used for the determination of the apparent rate constant (kapp). The values of kapp are −0.00157, −0.00414, −0.00209, −0.0024 and −0.0048 min^−1^ for NiO_aq_, NiO_et_, NiO_aq_@GO, NiO_et_@GO and GO, respectively (Fig. S6 of Supplementary Information).

Statistical analysis was performed by using two-way ANOVA of the photocatalytic degradation efficiency of CV in the presence of NiO_aq_ (Fig. 15a), NiO_et_ (Fig. 15b), NiO_aq_@GO (Fig. 15c), NiO_et_@GO (Fig. 15d) and GO (Fig. 15e) which showed significant results (P < 0.05) represented in Table 4.

**Table 4:** Statistical analysis by two-way ANOVA for photodegradation efficiency of CV in the presence of NiO_aq_, NiO_et_, NiO_aq_@GO, NiO_et_@GO and GO.

Source of Variation	SS	Df	MS	F	P-value	F crit
Rows	2849.52	4	712.38	3.98	0.02	3.01
Columns	2235.91	4	558.98	3.12	0.04	2.98
Error	2865.63	16	179.10			

### 3.9 Comparison of results with previous literature

According to the literature (Table 5), when NPs are synthesized using various plant extracts, the size of Ni/NiO nanoparticles typically ranges from 1 to 100 nm, while NiO nanoparticles range from 8 to <100 nm. These studies indicate that in most cases, Ni/NiO exhibit face-centered cubic or spherical structures, rhombohedral, and crystalline, while a few cases show octahedral, triangular, or agglomerated forms. Additionally, previous research investigated the antibacterial, antifungal, anticancer, antioxidant, cytotoxic, hemolytic, enzymatic inhibition, reducing power, catalytic, and optical properties of NiO nanoparticles synthesized using green methods (Table 5). However, no electrochemical studies of *E. cardamomum* mediated NiO NPs were reported earlier. The synthetic materials and important characteristics of the investigated NiO nanoparticles were compared to the previously reported Ni/NiO NPs, and the results are summarized in Table 5.

**Table 5:** Properties of nickel/nickel oxide nanoparticles in various plant extracts.

Sr. No.	Plant extract	Nickel salt solution used	Form, size and shape of nanoparticles	Applications tested	Reference
1	*E. cardamom* leaves	Ni(NO_3_)_2_.6H_2_O	NiO_aq_ (30.63 nm) and NiO_et_ (27.7 nm) Spherical; NiO_aq_@GO (26.39 nm) and NiO_et_@GO (19.33 nm) Randomly agglomerated	Electrochemical and photocatalytic degradation of dye	Current Study
2	Aqueous extract of *Azadirachta indica* and *Psidium guajava* leaves	Nickel chloride (NiCl_2_)	NiO = 22 nm Ni = 44 nm Pure cubic face-centered	Cytotoxic effect on the HT29 cell lines	[45]
3	Extract of *Apium graveolens*	Nickel acetate (Ni(CH_3_COO)_2_)	Ni = 6–45 nm Cubic	Elimination of aliphatic hydrocarbons from crude oil	[46]
4	*Camellia sinensis* leaves extract	Nickel chloride hexahydrate (NiCl_2_·6H_2_O) in the presence of pure water	Ni = 43.87–48.76 nm Uniform and spherical	Photocatalytic effect under solar light irradiation	[47]
5	Leaves of *Euphorbiamaculata*	Nickel chloride hexahydrate (NiCl_2_·6H_2_O)	NiFe_3_O_4_ = 30 nm Crystalline	Dye removal efficiency	[48]
6	Extract of Sundried *Alfalfa*	Nickel nitrate (Ni(NO_3_)_2_)	Ni = 1–6 nm and statistic size 3.4 ± 0.9 nm Spherical	Used for bioreduction by utilizing flavonoids and reducing sugars	[49]
7	Leaves extract of *Fumaria officinalis*	Nickel sulphate hexahydrate (NiSO_4_·6H_2_O) in the presence of NaOH	Ni = 16.85–49.04 nm Spherical	Anti-human ovarian cancer	[50]
8	Leaves extract of *Calotropis gigantean*	Nickel nitrate hexahydrate (Ni(NO_3_)_2_·6H_2_O) in the presence of NaOH, methanol and sodium borohydride	Ni/NiO, Ni = 20–40 nm, NiO = 60 nm Spherical	For antimicrobial potential, degradation of methylene blue	[51]
9	*Ocimum sanctum* leaves extract	Nickel nitrate (Ni(NO_3_)_2_) in the presence of barium sulphate and potassium nitrate	Ni = between 12 and 36 nm Spherical	As adsorbent of hazardous anionic pollutants and dyes	[52]
10	*Phlomis cancellate bunge* leaves extract	Nickel nitrate (Ni(NO_3_)_2_)	Ni = 15–25 nm Spherical	Photocatalytic degradation	[53]
11	Through solvothermal path using EG	Nickel chloride hexahydrate (NiCl_2_·6H_2_O) in the presence of PEG	Ni = 24–49 nm Crystalline	To improve the mechanical, thermal, and electrical characteristics of polymeric materials	[54]
12	*Monsonia burkeana* leaves extract	Nickel nitrate hexahydrate (Ni(NO_3_)_2_·6H_2_O) in the presence of ethyl glycol, citric acid	NiO = 20 nm Spherical	Usage for potential drug delivery vehicles against human cancers, photocatalytic activities	[55]
13	Leaf extract of *Ageratum conyzoides* L.	Nickel nitrate (Ni(NO_3_)_2_·6H_2_O) in the presence of methanol, n-hexane	NiO = 8–15 nm Cubic	Catalytic activity in the reduction of methylene blue	[56]
14	*Moringa oleifera* leaves extract	Nickel nitrate (NiNO_3_)_2_	NiO = 9.69 nm Spherical or cylindrical	*In-vitro* cytotoxicity and antibacterial activities	[57]
15	*Rhamnus triquetra* leaves extract	Nickel nitrate (Ni(NO_3_)_2_)	NiO = approx 25 nm agglomerated/spherical	*In-vitro* potentials such as anticancer, antileishmanial activities	[58]
16	Roots extract of *Desmodium gangeticum*	Nickel chloride (NiCl_2_) in the presence of NaOH	Ni = 6.04 nm Spherical	Antibacterial activities were performed	[59]
17	Leaves extract of *Pulmonaria longifolia* and *Ficus religiosa*	Nickel chloride (NiCl_2_)	Ni = 42.21 nm and 43.178 nm Spherical	For chromium removal	[60]
18	*Lactuca serriola* seeds extract	Nickel chloride hexahydrate (NiCl_2._6H_2_O)	Ni/NiO = < 100 nm Spherical	For environmental remediation and antibacterial applications	[61]
19	*Geranium wallichianum* leaves extract	Nickel nitrate (Ni(NO_3_)_2_)	NiO = approximately 21 nm Spherical	Antibacterial, antifungal and antileishmanial activities	[62]
20	Leaves extract of *Alhagi maurorum*	Nickel sulphate hexahydrate (NiSO_4_.6H_2_O) in the presence of NaOH	Ni = 20.56–36.63 nm Spherical	Cytotoxicity and anti-human ovarian cancer effect	[63]
21	Stem extract of *Berberis balochistanica*	Nickel nitrate (Ni(NO_3_)_2_)	NiO = 31.44 nm Rhombohedral	Antioxidant properties	[64]
22	*Salvia hispanica* seed extract	Nickel nitrate hexahydrate (NiNO_3_)_2_·6H_2_O	NiO = 30 nm Spherical	Cytotoxicity effect and photocatalytic activities	[65]
23	Leaves extract of *Aegle marmelos correa*	Nickel chloride hexahydrate (NiCl_2._6H_2_O)	Ni = 80–100 nm Triangular	Mosquito larvicidal and anti-inflammatory activities	[66]
24	*Vernonia amygdalina* leaves extract	Nickel chloride hexahydrate (NiCl_2._6H_2_O)	NiO = 17.86 nm Octahedral	Antimicrobial activities	[67]
25	Leaves extract of *Andrographis paniculata*	Nickel acetate tetrahydrate (Ni(CH_3_COO)_2_.4H_2_O)	NiO = 24 nm Spherical	Anti-cancer and photocatalytic activities	[68]
26	*Sageretia thea* leaves extract	Nickel nitrate (Ni(NO_3_)_2_)	NiO ≈ 18 nm Spherical	Antioxidant, *In-vitro* pharmacognostic and cytotoxic potential	[69]
27	Shell waste of *Prunus dulcis*	Ni(NO_3_)_2_.6H_2_O	Amorphous	Electrochemical potential	[70]

## 4 Conclusions

The rapid, cost-effective, sustainable, and environmentally friendly nature of NPs synthesis has captured the interest of researchers. We have established an eco-friendly method for the production of NiO NPs (NiO_aq_ and NiO_et_) from Ni(NO_3_)_2_.6H_2_O by treating it with aqueous/ethanolic extracts of *E. cardamomum* leaves. The same reaction was performed in the presence of graphene oxide to produce NiO_aq_@GO and NiO_et_@GO NCs. The synthesized NMs were subjected to characterization by XRD, FT-IR, SEM, EDX, UV–visible spectroscopy, and TGA-DSC analyses and were also evaluated for their electrochemical properties and photocatalytic degradation of crystal violet dye. XRD analysis revealed the average crystallite sizes of 8.84–14.07 nm of NMs with a face-centered cubic form of NiO NPs and a hexagonal structure of their nanocomposites with GO. FT-IR spectroscopy confirmed the presence of Ni-O vibrations in the range of 443-436 cm^−1^. SEM images confirmed the spherical morphology of NiO NPs while NiO_aq_@GO NCs contained randomly aggregated, thin, and wrinkled graphene sheets. The particle sizes of NiO_aq_, NiO_et_, NiO_aq_@GO, NiO_et_@GO and GO were found to be 30.63, 27.7, 26.39, 19.33 and 43.7 nm, respectively. The EDX spectra showed the homogeneous distribution of elements. The UV–visible spectroscopy revealed the band gaps of 5.0, 3.89, 3.74, 3.34, and 4.48 eV for NiO_aq_, NiO_et_, NiO_aq_@GO, NiO_et_@GO, and GO, respectively. TGA-DSC was used to calculate the weight losses, enthalpies (normalized) and glass transition temperatures of all the NMs. The thermal decomposition patterns of NiO_aq_ and NiO_et_ were entirely different as compared to those of NiO_aq_@GO, NiO_et_@GO, and GO. Cyclic voltammetry (CV) measurements were conducted at various scan rates within a potential range of −0.5-0.5 V, which have shown the prominent oxidation and reduction peaks. The synthesized NCs hold potential for future applications as electrode materials in supercapacitors. The NMs were also tested for photocatalytic degradation of crystal violet (CV) dye; the degradation coefficient of CV were found to be 82.93, 86.34, 89.99, 90.27, and 81.65 % in the presence of NiO_aq_, NiO_et_, NiO_aq_@GO, NiO_et_@GO, and GO, respectively after 120-min exposure to sunlight. Current study is significant in view of its eco-friendly bio-synthetic approach for the production of NiO NPs and their nanocomposites with graphene oxide (GO). It also focuses on the electrochemical performance of NPs as electrode materials and their photodegradation efficiency for the removal of crystal violet dye from wastewater. This study may possibly be extended to photocatalytic degradation of many other dyes, and the potential uses of synthesized NMs in hydrogen evolution reaction, energy storage devices, and as electrode materials for batteries and solar cells. In the investigated work, biosynthesis of NiO nanoparticles demonstrated challenges including aggregation (noted in NiOaq/GO composites), inconsistent crystalline sizes, high band gaps, limiting photocatalytic efficiency and less yields of products. Future work should focus on improving stability, charge separation, and environmental safety.

## Funding

This research was funded by Princess Nourah bint Abdulrahman University Researchers Supporting Project number (PNURSP2024R439), Princess Nourah bint Abdulrahman University, Riyadh, Saudi Arabia.

## CRediT authorship contribution statement

**Ayesha Kiran:** Writing – original draft, Methodology, Investigation. **Shabbir Hussain:** Writing – review & editing, Supervision, Conceptualization. **Israr Ahmad:** Validation, Conceptualization. **Muhammad Imran:** Writing – review & editing, Validation, Project administration. **Muhammad Saqib:** Writing – review & editing. **Bushra Parveen:** Project administration, Formal analysis. **Khurram Shahzad Munawar:** Investigation, Formal analysis. **Wissem Mnif:** Resources, Funding acquisition. **Maryam Al Huwayz:** Funding acquisition, Resources, Investigation. **Norah Alwadai:** Formal analysis, Data curation. **Munawar Iqbal:** Writing – review & editing.

## Declaration of competing interest

The authors declare that they have no known competing financial interests or personal relationships that could have appeared to influence the work reported in this paper.
