# Supplementary material for: Green synthesis of NiO and NiO@graphene oxide nanomaterials using Elettaria cardamomum leaves: Structural and electrochemical studies
Source: Heliyon. 2024 Sep 30;10(20):e38613. doi: 10.1016/j.heliyon.2024.e38613 (PMC11497386; doi:10.1016/j.heliyon.2024.e38613)
Supplement: Multimedia component 1 [file mmc1.docx]

**Green Synthesis of NiO and NiO@ Graphene Oxide Nanomaterials using *Elettaria cardamomum* Leaves: Structural and Electrochemical Studies**

Ayesha Kiran^1^, Shabbir Hussain^1,^*, Israr Ahmad^1^, Muhammad Imran^2^, Muhammad Saqib^1^, Bushra Parveen^3^, Khurram Shahzad Munawar^4,5^, Wissem Mnif^4,6^, Maryam Al Huwayz^4,7^, Norah Alwadai^4,7^, Munawar Iqbal^8^

^1^Department of Chemistry, Khwaja Fareed University of Engineering and Information Technology, Rahim Yar Khan, Pakistan

^2^Division of Inorganic Chemistry, Institute of Chemistry, The Islamia University of Bahawalpur, 63100 Bahawalpur, Pakistan

^3^Department of Chemistry, Government College University Faisalabad, Pakistan

^4^Institute of Chemistry, University of Sargodha, 40100, Pakistan

^5^Department of Chemistry, University of Mianwali, 42200, Pakistan

^6^Department of Chemistry, Faculty of Sciences at Bisha, University of Bisha, P.O. BOX 199, Bisha 61922, Saudi Arabia

^7^Department of Physics, College of Sciences, Princess Nourah bint Abdulrahman University, P.O. Box 84428, Riyadh 11671, Saudi Arabia

^8^School of Chemistry, University of the Punjab, Lahore 54590, Pakistan

*Corresponding Author: [shabchem786@gmail.com](mailto:shabchem786@gmail.com); [syedadnan@uitm.edu.my](mailto:syedadnan@uitm.edu.my)

**Supplementary Information**

**
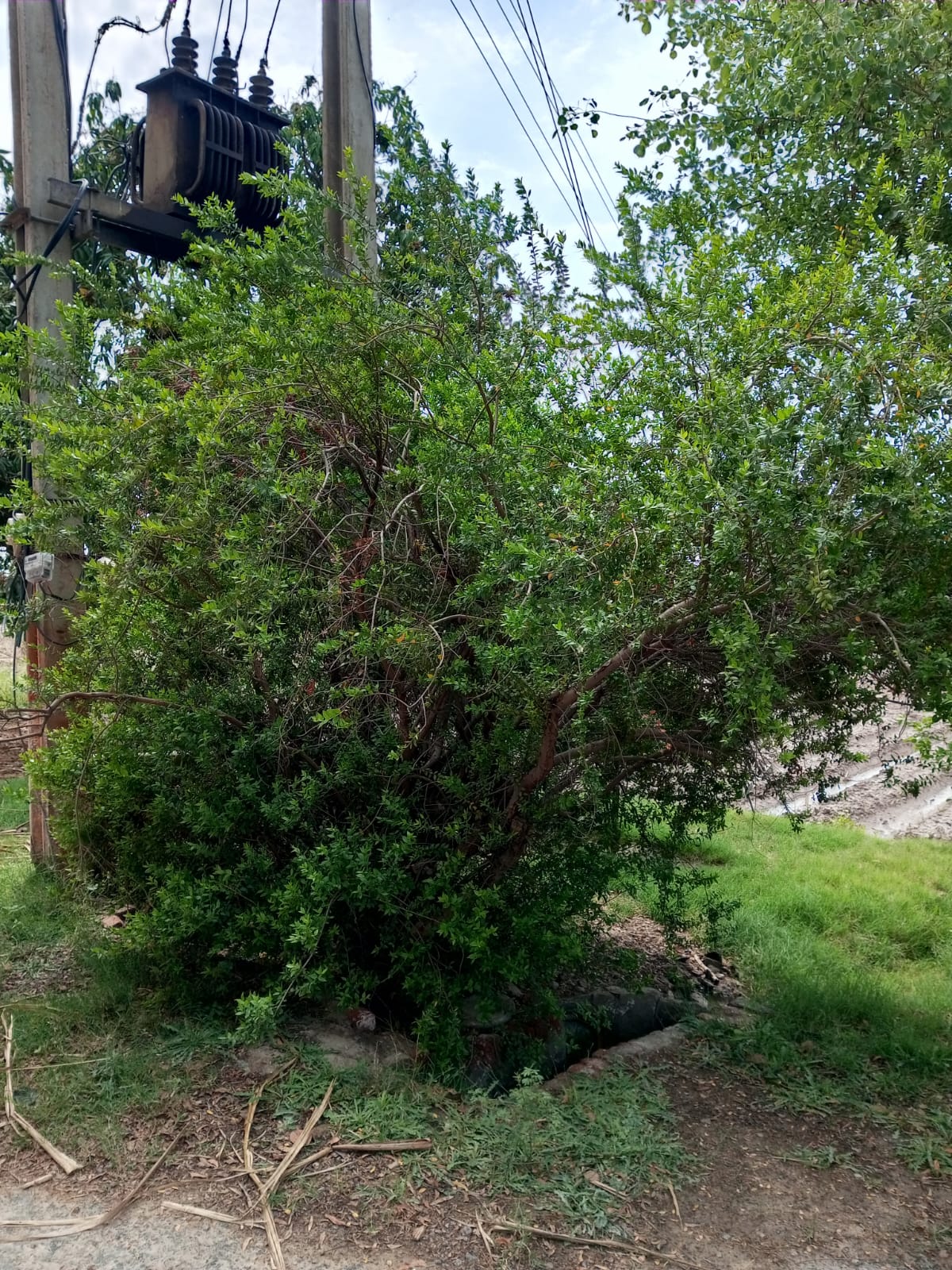

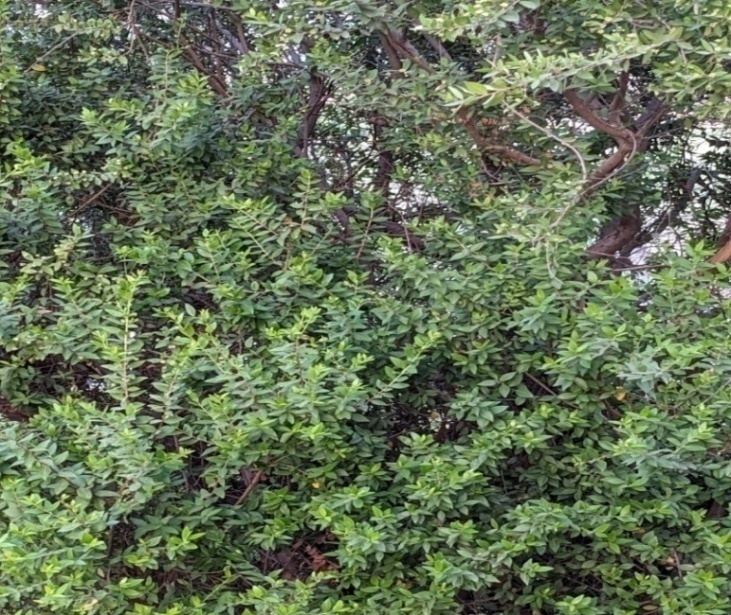
**

**Fig. S1.** *E. cardamomum* plant used for the synthesis of *nickel oxide* (NiO) nanoparticles


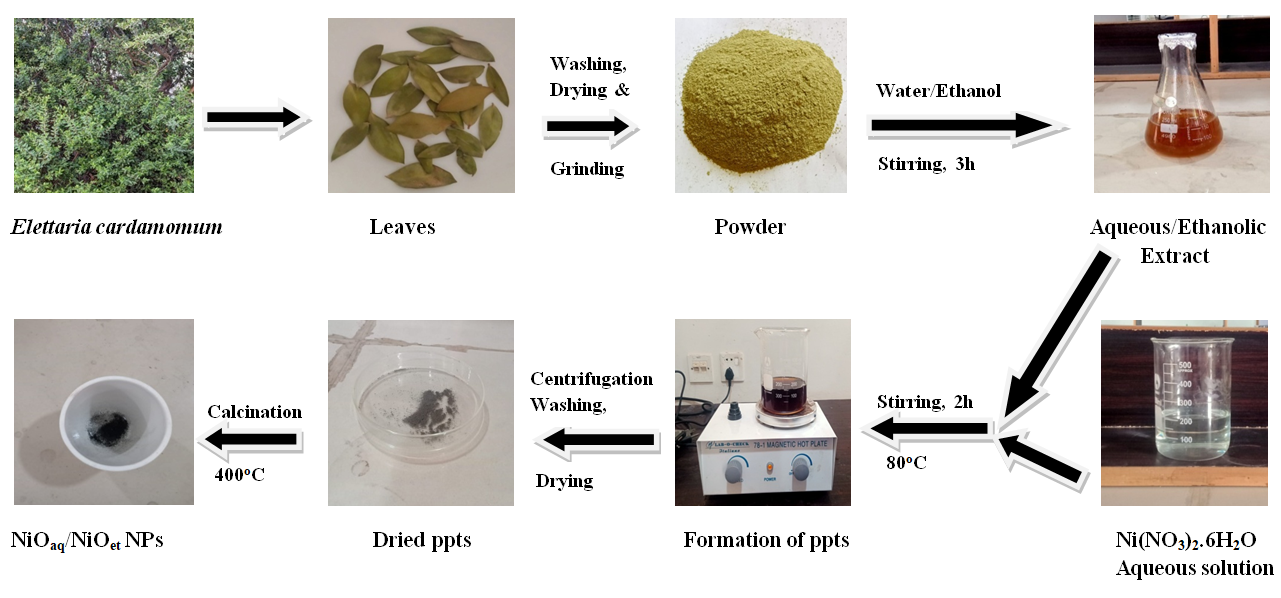


**Fig. S2.** E. cardamomum mediated synthetic route for nickel oxide (NiO_aq_ & NiO_et_) NPs


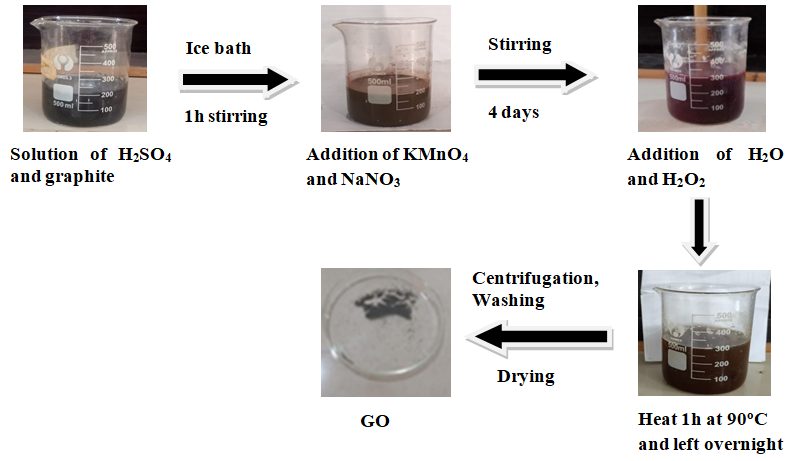


**Fig. S3.** Synthetic route for graphene oxide (GO)


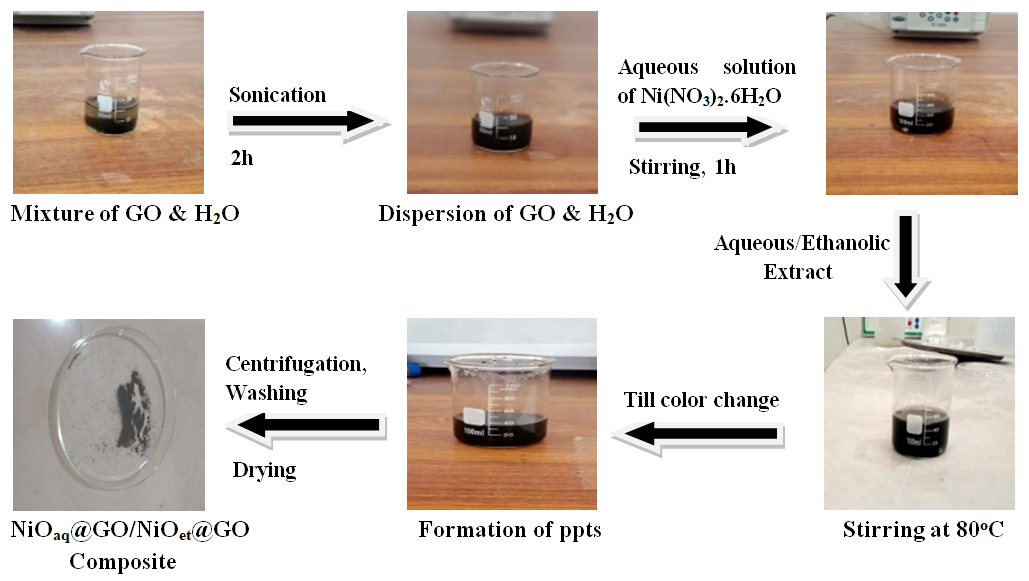


**Fig. S4.** E. cardamomum mediated synthetic route for NiO_aq_@GO and NiO_et_@GO nanocomposites

**Fig.** S5. Absorbance spectra of CV dye in the presence of NiO_aq_ (a), NiO_et_ (b), NiO_aq_@GO (c), NiO_et_@GO (d) and GO (e)

**Fig. S6.** Kinetic behavior of photodegradation of CV dye in the presence of NiO_aq_(a),NiO_et_(b),NiO_aq_@GO(c),NiO_et_@GO(d) and GO (e)
